# Tissue-specific transcriptome assemblies of the marine medaka Oryzias melastigma and comparative analysis with the freshwater medaka Oryzias latipes

**DOI:** 10.1186/s12864-015-1325-7

**Published:** 2015-02-27

**Authors:** Keng Po Lai, Jing-Woei Li, Simon Yuan Wang, Jill Man-Ying Chiu, Anna Tse, Karen Lau, Si Lok, Doris Wai-Ting Au, William Ka-Fai Tse, Chris Kong-Chu Wong, Ting-Fung Chan, Richard Yuen-Chong Kong, Rudolf Shiu-Sun Wu

**Affiliations:** School of Biological Sciences, Kadoorie Biological Sciences Building, The University of Hong Kong, Pokfulam Road, Hong Kong, SAR China; School of Life Sciences, Hong Kong Bioinformatics Centre, The Chinese University of Hong Kong, Hong Kong, SAR China; Genome Research Centre, The Hong Kong Jockey Club Building for Interdisciplinary Research, The University of Hong Kong, 5 Sassoon Road, Pokfulam, Hong Kong, SAR China; Department of Biology and Chemistry, City University of Hong Kong, Tat Chee Avenue, Kowloon, Hong Kong, SAR China; Department of Biology, Hong Kong Baptist University, Hong Kong, SAR China; The State Key Laboratory in Marine Pollution, Hong Kong, China

**Keywords:** Medaka, Marine and freshwater, Transcriptome assembly, *De novo* assembly, High-throughput RNA sequencing

## Abstract

**Background:**

The marine medaka *Oryzias melastigma* has been demonstrated as a novel model for marine ecotoxicological studies. However, the lack of genome and transcriptome reference has largely restricted the use of *O. melastigma* in the assessment of *in vivo* molecular responses to environmental stresses and the analysis of biological toxicity in the marine environment. Although *O. melastigma* is believed to be phylogenetically closely related to *Oryzias latipes*, the divergence between these two species is still largely unknown. Using Illumina high-throughput RNA sequencing followed by *de novo* assembly and comprehensive gene annotation, we provided transcriptomic resources for the brain, liver, ovary and testis of *O. melastigma*. We also investigated the possible extent of divergence between *O. melastigma* and *O. latipes* at the transcriptome level.

**Results:**

More than 14,000 transcripts across brain, liver, ovary and testis in marine medaka were annotated, of which 5880 transcripts were orthologous between *O. melastigma and O. latipes*. Tissue-enriched genes were identified in *O. melastigma*, and Gene Ontology analysis demonstrated the functional specificity of the annotated genes in respective tissue. Lastly, the identification of marine medaka-enriched transcripts suggested the necessity of generating transcriptome dataset of *O. melastigma*.

**Conclusions:**

Orthologous transcripts between *O. melastigma* and *O. latipes*, tissue-enriched genes and *O. melastigma*-enriched transcripts were identified. Genome-wide expression studies of marine medaka require an assembled transcriptome, and this sequencing effort has generated a valuable resource of coding DNA for a non-model species. This transcriptome resource will aid future studies assessing *in vivo* molecular responses to environmental stresses and those analyzing biological toxicity in the marine environment.

**Electronic supplementary material:**

The online version of this article (doi:10.1186/s12864-015-1325-7) contains supplementary material, which is available to authorized users.

## Background

There is a trend of using small marine fish as models to study the biological impact of environmental pollutants and stresses on marine organisms, which is an important area of ecotoxicological studies [1]. Freshwater fish models, such as zebrafish (*Danio rerio*) and rainbow trout (*Oncorhynchus mykiss*), have been widely used for ecotoxicological studies in the freshwater environment. However, their responses to environmental toxins can be completely different in marine fish [2-4]. For example, it has been reported that freshwater species were more sensitive to ammonia and metal compounds whereas marine species were more sensitive to pesticide and narcotic compounds [4]. Such differences indicate that ecotoxicological results from freshwater environments cannot be directly applied to the marine environment [1]. The marine medaka *Oryzias melastigma* (*O. melastigma*) is an emerging marine fish model used in the investigation of the response of organisms to pollutants, toxins and stresses in marine environments [5,6]. In fact, *O. melastigma* is already used in a variety of estuarine and marine ecotoxicological studies [7-10], demonstrating their potential in studying the effect of organic chemicals, inorganic chemicals, microorganism and environmental stresses in relation to cardiac toxicity [11], hepatotoxicity [9], neurotoxcity [12], immunotoxicity [10], and so forth. In addition, *O. melastigma* has been adopted by the International Life Sciences Institute (ILSI) Health and Environmental Science Institute (HESI) for embryo toxicity testing. Unfortunately, the use of *O. melastigma* as a model in the assessment of *in vivo* molecular responses to environmental stresses and for analyzing biological toxicity in the marine environment is largely restricted by the lack of molecular resources for *O. melastigma* [13].

*O. melastigma* was previously believed to be phylogenetically closely related to the Japanese freshwater ricefish medaka *Oryzias latipes* (*O. latipes*) [1,14], of which a draft genome has been reported [15]. However, even within inbred strains within the *O. latipes* species group, the genome-wide SNP rate between the Hd-rR and HNI strains is among the highest (3.42%) of all vertebrate species [15]. Recently, *O. melastigma* and *O. latipes* were shown to belong to two distinct species groups of medaka [16], suggesting they could be even more divergent. Therefore, there may be a pressing need of a genetic database specifically devoted for the marine medaka *O. melastigma*.

Here, using Illumina high-throughput RNA sequencing (RNA-Seq) followed by *de novo* assembly and comprehensive annotation and comparison of the transcriptome dataset, we provide transcriptomic resources, including the brain, liver and gonadal tissues (ovary and testis) of female and male *O. melastigma*. Our primary goal was to produce a reference set of mRNA sequences for *O. melastigma* that would facilitate the understanding of the local adaptation, genome evolution and population genetics of medaka. Additionally, the identification of a set of genes along with their functional annotation in multiple organs of *O. melastigma* would facilitate the use of marine medaka for ecotoxicology studies. Furthermore, we compared the gene sets of *O. melastigma* and *O. latipes* to determine their possible divergence at the transcriptomic level.

## Methods

**Figure 1:**
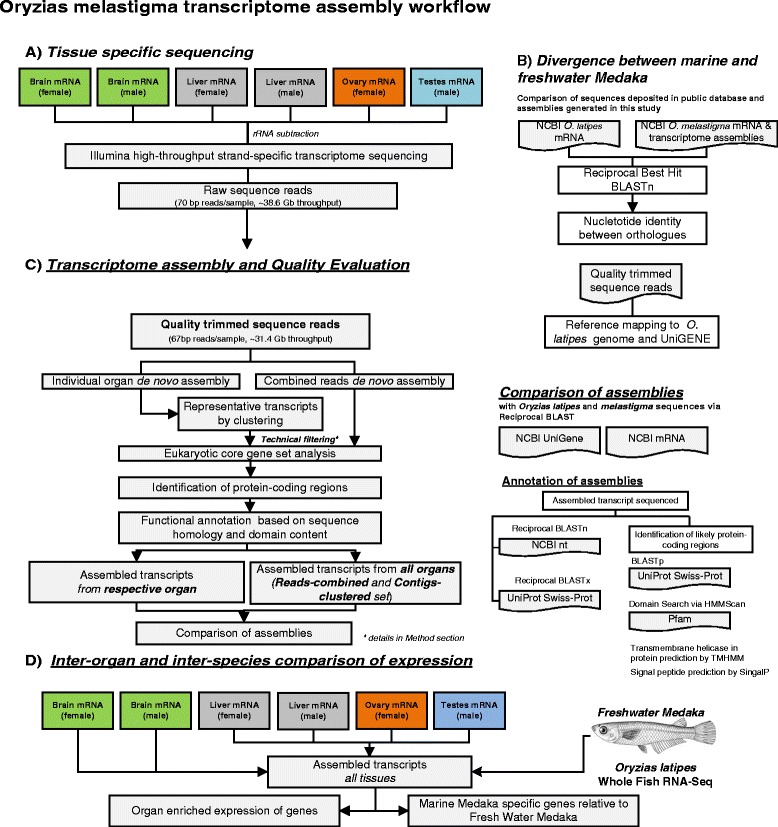
**Workflow of this study. A)** Organ-specific transcriptome sequencing using the Illumina GAIIx platform. **B)** Overview of the procedure to investigate the divergence between marine and freshwater medaka at the transcriptome level. **C)** Pipeline of *de novo* transcriptome assembly. Refer to main text for details. **D)** Comparison of inter-organ transcription and marine-to-freshwater medaka.

Tissue specific transcriptome from of *O. melastigma* were assembled from high-throughput strand-specific RNA-Seq. The possible divergence between marine and freshwater medaka at the transcriptome level was assessed by comparisons of sequences deposited in public databases and the assemblies generated in this study. A single consensus transcriptome was generated for gene annotation and inter-organ comparative analysis and marine-to-freshwater medaka transcriptome comparison. The overall workflow of the study is shown in Figure 1.

### Medaka maintenance and RNA isolation

All animal research procedures were approved by the Committee on the Use of Live Animals in Teaching and Research (CULATR) at The University of Hong Kong. The freshwater medaka fish *O. latipes* was gifted by David Hinton's laboratory at Duke University. Marine medaka (*O. melastigma*) were reared and maintained under optimal growth and breeding conditions, as described in Kong *et al*. (5.8 mg O_2_ L^−1^, 28 ± 2^o^ C, pH 7.2 in a 14-h light: 10-h dark cycle) [1]. The stock of marine medaka used in our experiment was obtained from Interocean Industries (Taiwan) and has been reared in our laboratory for over 10 generations. 1:1 ratio of sexually mature 4-month old male and female medaka were placed in a same tank for external fertilization to take place naturally and the fertilized eggs were collected [17]. At 120 days post fertilization, brain, liver, ovary and testis tissues were dissected from randomly selected male (*n* = 10) and female (*n* = 10) fish. To minimize the variation among individual fish, tissue samples from 10 fish were pooled. Total RNA from pooled tissue samples was extracted using the mirVana^TM^ isolation kit (Applied Biosystems) and then treated with DNase (Ambion) to remove contaminating genomic DNA. The RNA quality was assessed using the Agilent 2100 Bioanalyzer system, and samples with an RNA Integrity Number (RIN) greater than 9 were used for RNA library construction.

### Strand-specific library preparation and transcriptome sequencing

Sequencing was performed at the Centre for Genomic Sciences, The University of Hong Kong. Total RNA was treated with the RiboMinus Eukaryote Kit for RNA-Seq (Invitrogen, Carlsbad, CA) to remove ribosomal RNA, and the rRNA-depleted RNA was concentrated by ethanol precipitation in the presence of a glycogen carrier (Ambion). The dUTP strand-specific library construction protocol was used to generate templates for Illumina DNA sequencing. Briefly, strand specificity was maintained by the incorporation of deoxy-UTP during second-strand cDNA synthesis and subsequent destruction of the uridine-containing strand during the following step of library construction. The sequencing library was constructed using GAIIx with the use of the Paired-End Cluster Generation Kit v5 and Sequencing Kit v5 (Applied Biosystems) following the manufacturer’s recommended protocol, which generated 76-bp-long paired-end sequence reads. The insert size was approximately 200 bp.

### Transcriptome assembly

The sequence reads were dynamically trimmed according to BWA’s –q algorithm with a parameter of 30. A running sum algorithm was executed. Briefly, an cumulative area plot is plotted from 3’-end to the 5’-end sequence reads, where positions of base-calling Phred quality lower than 30 causes an increase of area and vice versa. Such plot was built for each read individually. The read would be trimmed from the 3’-end to the position where the area was greatest [18]. Read pairs were then synchronized such that all read pairs with sequences of at least 35 bp on both sides after quality trimming were retained and any singleton read resulted from reads trimming were removed. The quality-trimmed sequence reads were assembled using Trinity (r2013-02-25) [19], which uses fixed *k*-mer to generate assembly and is efficient in recovering full-length transcripts and spliced isoforms [19]. Trinity was used rather than multi *k*-mer tools because Trinity was shown to reconstruct the most full-length transcripts for genes expressed in different dynamic ranges when compared with the various single *k*-mer assemblers, while multi *k*-mer tools tended to assemble more artificially fused transcripts [20]. *De novo* assembly by Trinity was individually performed for each organ and gender. For brain and liver, an additional gender-pooled *de novo* sequence read assembly was performed. Such gender-pooled assemblies were used to facilitate comparison of tissue enriched genes based on annotation of the assembled transcripts (section Tissue-enriched genes in O. melastigma). Assembled transcripts from individual samples were merged and duplicates were then removed using CD-hit-est [21] (v4.5.4) using the accurate mode (−g 1) with other parameters left as default to yield the final assembly (Contigs-clustered Assembly). CD-Hit uses an incremental clustering algorithm to first sort all assembled transcripts in order of decreasing length. The longest transcript becomes the representative of the first cluster. Then, each remaining transcript is compared to the representatives of all existing clusters and would be clustered to the most similar cluster if the similarity is above threshold of global sequence identity of ≥ 90%. Otherwise a new cluster is defined with that sequence being the representative [22]. Such a merging process broadens the coverage of assemblies produced by Trinity. A *de novo* meta-tissue assembly (Reads-combined Assembly) [23] was also performed using a virtual library by merging sequence reads from all organs (see also discussion below and Figure 1).

### Assembly validation and transcript annotation

We employed an internal validation approach for mapping quality-trimmed sequence reads back to the assembly to identify poor-quality and potentially misassembled transcripts. Through the process, transcripts with an average base coverage of less than one were removed from the assembly sets. The quality of the assembled transcripts was then assessed using the metric that was suggested for *de novo* transcriptome assembly [24], including contig count, percentage of reads used in contig, base-pairs in contig, average contig coverage, average contig length and contig N50 length. The quality of the assembly was further assessed by comparison with the 248 core eukaryotic genes (CEGs) [25] with the use of BLASTp, an e-value cut-off of 1.0x10^−6^ [26,27] and a requirement of more than 70% alignment length for the CEGs.

In the first step of transcript annotation, the assembled transcripts were compared to (1) the NCBI non-redundant nucleotide (nt) database with the use of Reciprocal BLASTn; and (2) the UniProt Swiss-Prot protein database with the use of Reciprocal BLASTx. Orthologs were identified if they were the symmetrical best hits in each reciprocal all-against-all (i.e., Reciprocal Best Hit) in the BLASTn and BLASTx search [28]. Briefly, orthologs to the sequences in the nt and Swiss-Prot databases were identified first by BLASTing the assembled transcript to the database. The highest-scoring hit was obtained and then BLASTed against the database of the assembled transcripts. The hit in the nt and Swiss-Prot databases was considered an ortholog of the assembled transcript if and only if the second BLAST returned the assembled transcript that was the highest scorer in the first BLAST.

As an alternative approach to annotate the assembled transcripts, protein-coding regions within the transcripts were first identified using the TransDecoder algorithm [23]. Briefly, 500 of the longest Open Reading Frames (ORFs) were extracted and used to build a Markov model based on hexamers. These likely coding sequences were randomized to provide a sequence composition corresponding to a non-coding sequence. All of the longest ORFs in each of the six possible reading frames were scored according to the Markov Model (log likelihood ratio based on coding/noncoding). If the proper coding frame of the putative ORF scored positive and was the highest of the other presumably wrong reading frames, then that ORF was reported. If a high-scoring ORF was eclipsed by (fully contained within the span of) a longer ORF in a different reading frame, it was excluded. The likely protein-coding regions were then subjected to (1) BLASTp searching against UniProtKB/Swiss-Prot with an e-value cut-off of 1.0x10^−6^ [26,27], (2) a protein domain search via HMMScan, (3) transmembrane helicase prediction by TMHMM and (4) signal peptide prediction by SignalP.

### Discovery of tissue-enriched and O. melastigma-enriched genes

An annotation-based approach was used to discover the tissue-enriched genes of *O. melastigma*. Quality-filtered transcripts with Reciprocal Best Hits (nt database and UniProt) were considered. For the brain and liver, of which both male and female transcriptomes were sequenced, matches to annotations were merged, and a union set was used. To compare the transcriptome between *O. melastigma* and its freshwater counterpart *O. latipes*, we obtained 2 independent sets of whole-fish, deep RNA-Seq data from the NCBI Sequence Read Archive (SRA) under Accession SRP004363 and SRP032993 and calculated the transcript expression based on our Reads-combined Assembly of the *O. melastigma* transcriptome. Briefly, *O. melastigma* transcripts with ≥ 8 reads, but without any read-count in both independent freshwater RNA-Seq datasets were considered to be putative *O. melastigma*-enriched transcripts. *O. melastigma*-enriched transcripts across a dynamic range of expression were then subjected to qPCR validation to determine the optimal read-count threshold. Since the *O. latipes* RNA-Seq dataset we retrieved from NCBI SRA were yet to be published, we only sought to discover *O. melastigma*-enriched genes with respective to *O. latipes*.

### qPCR validation in independent samples

Quantitative real-time PCR was used to detect the expression of select genes that are closely related to the functions of corresponding tissues, and 18S ribosomal RNA (*18S*) was used as reference gene for qPCR normalization. The primer sequences are listed in Additional file 1: Table S1. cDNA was synthesized from 1 μg of total RNA extracted from an independent set of medaka using the SuperScript® VILO™ cDNA Synthesis Kit (Life Technologies). The reverse transcription reactions were incubated in a C1000 Thermal Cycler (Bio-Rad) at 25°C for 10 min, 42°C for 60 min and 85°C for 5 min and then held at 4°C. qRT-PCR was performed using the StepOnePlus Real-Time PCR system (Applied Biosystems). The 20-μl PCR reaction included 1 μl of RT product, 10 μl of KAPA SYBR® FAST qPCR Master Mix (2X), 0.5 μl of each primer (10 μM), and 8 μl of nuclease-free water. The reactions were incubated in a 96-well optical plate at 95°C for 10 min, followed by 40 cycles at 95°C for 15 sec and 60°C for 1 min. Reactions were run in triplicate and included a no-template control for each gene. The relative expression ratio of *target*/*18S* was calculated according to the method described by Pfaffl [29]:

$$ \mathrm{Expression}\ \mathrm{ratio} = {{\mathrm{E}}_{target}}^{\mathrm{CP} target\left(\mathrm{control}\hbox{--} \mathrm{treatment}\right)}/\ {{\mathrm{E}}_{\mathit{\mathsf{18}}\mathit{\mathsf{S}}}}^{\mathrm{CP}\mathit{\mathsf{18}}\mathit{\mathsf{S}}\;\left(\mathrm{control}\hbox{--} \mathrm{treatment}\right)}, $$

where E = 10^(–1/slope)^ and CP is the crossing point at which fluorescence rises above background. Statistical significance was calculated using the Wilcoxon–Mann–Whitney test.

### Genome reference, genomic resources and tools used

The medaka HdrR reference genome v.72.1 was retrieved from Ensembl [30], and the RNA-Seq data of freshwater *O. latipes* were retrieved from NCBI SRA (SRP004363 and SRP032993). STAR aligner [31] was used to align the transcriptome data to the genome, and reference mapping of the *O. latipes* UniGENE and RNA-Seq datasets to the assembled transcript re-mapping was performed using BWA-MEM v.0.7.5a-r405 and Novoalign v3.00.05 (http://www.novocraft.com/). Gene Ontology enrichment (Biological Process, Cellular Component, and Molecular Function) was performed using BinGO [32], which is implemented in Cytoscape (http://www.cytoscape.org/).

## Results and discussion

Transcriptome sequencing of 4 organs (brain, liver, ovary and testis) of male and female *O. melastigma* in 6 libraries yielded 34.81 Gbp of mRNA sequences from approximately 505 million ~70-bp paired-end reads (average 84 million reads per tissue). The coverage for each library was more than 100-fold based on the transcriptome size of the freshwater counterpart *O. latipes*. A previous study suggested that such sequencing depth, coupled with stringent sequence reads quality filtering, is optimal for tissue specific transcriptome assembly [33]. Four hundred and twenty-two million quality-trimmed reads, corresponding to 28.5 Gbp were subjected to downstream analysis. The sequencing statistics and technical details are shown in Additional file 2: Table S2.

### Comparison between the transcriptome of freshwater and marine medaka

**Figure 2:**
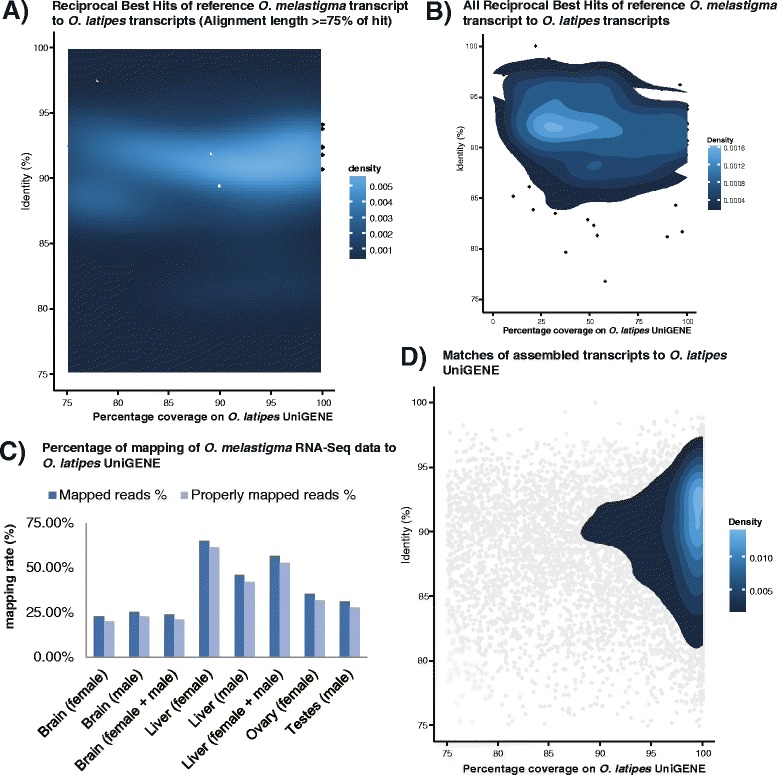
**Transcriptome divergence between marine and freshwater medaka. A)** Nucleotide identity between *Oryzias melastigma* and *Oryzias latipes* orthologs deposited in the NCBI nucleotide database. Only those with an alignment length >75% are shown. **B)** Nucleotide identity between all *O. melastigma* and *O. latipes* orthologs. **C)** Low mapping rate of *O. melastigma* RNA-Seq data generated in this study onto the *O. latipes* UniGENE dataset. The mapping rate is highest for liver and lowest for brain. **D)** The nucleotide identity between transcripts (Ensemble Assembly) assembled in this study and the *O. latipes* UniGENE dataset. Only those with alignment length >75% are shown.

In order to estimate the divergence of the *O. melastigma* and *O. latipes* transcriptomes, we first assessed their average nucleotide identities at the transcript level. Based on the mRNA transcripts deposited in the NCBI nucleotide database, orthologs in *O. melastigma* and *O. latipes* were identified using Reciprocal BLAST. The reciprocal best hit (RBH) was found for 58.6% (211/360) of the *O. melastigma* transcripts. Among the RBHs, the average identity was 91.5% (median: 91.8%), suggesting an extensive diversity between the two species. (Figure 2A-B). In line with our observation, phylogeographic studies of medaka using allozymes and mitochondrial DNA sequences have revealed a genetic diversity in the *Oryzias* family [34-36]. The studies showed that wild populations of medaka were divided into four major regionally differentiated groups and the Nei’s genetic distances among these groups are very large (0.35-0.88).

Using our RNA-Seq data, we then assessed if the Hd-rR (*O. latipes*) reference genome was suitable for marine counterparts, such as *O. melastigma*. For the two independent *O. latipes* RNA-Seq experiments, the majority of sequence reads (84.6-99.3%) could be aligned onto the Hd-rR reference genome (mismatch rate: 0.43-0.48%; unique aligned: 70.2-81.4%). However, when the *O. melastigma* quality-trimmed reads were aligned to the Hd-rR genome, the mapping rate ranged from only 38.4 to 52.3% (mismatch rate: 4.6-5.7%). Similarly, only a minority (22.8-65.0%) of reads could be aligned onto the *O. latipes* UniGENE dataset, meaning that our *O. melastigma* RNA-Seq data comprises a significant portion of transcribed sequences that could not be unaligned and thus might be absent in the existing freshwater medaka genome and transcriptome sequences. Thus, the current *O. latipes* sequences might not be suitable for genome-wide expression studies of *O. melastigma* (Figure 2C and Additional file 3: Table S3).

Our observations were not surprising and were in fact in line with previous finding suggesting that within the *O. latipes* species, the genome-wide SNP rate between the Hd-rR and HNI strains is highest (3.42%) among vertebrate species [15]. Such high divergence among different medaka species re-iterates that a marine transcriptome reference dataset, such as the emerging marine model *O. melastigma*, is imperative for studies that assess the responses of marine species to pollutants, toxins and stresses at the molecular level.

### Transcriptome assembly and generation of a consensus transcriptome

*De novo* assemblies of each library using Trinity resulted in an average of 85098 (51533–132296) contigs per sample. Brain tissue had the highest contig count, totaling approximately 15.6 Mbp. The lowest contig count was observed in liver tissue, with approximately 4.6 Mbp. The average contig length was 1106 bp and the contig N50 was 2162 bp. Nearly all transcripts (99.93%) had a coverage greater than 1 and were subjected to downstream annotation (Additional file 4: Table S4).

Using the transcriptome assemblies, we sought to rule out the possibility that the previously observed low-mapping rate of the *O. melastigma* RNA-Seq data onto the *O. latipes* UniGENE dataset was due to aligner bias. We assessed the recovery of the *O. latipes* UniGENE dataset based on our assembly result using BLASTn. With an e-value threshold of 1.0x10^−6^ [26,27], we found that, at most, 72.3% of the *O. latipes* UniGENE dataset (45.6% if ≥70% of the UniGENE dataset must be covered in terms of transcript length) could be matched to our assemblies, with an average identity, in bases, between transcripts of the two species of 89.6% (Figure 2D and Additional file 5: Table S5). In other words, the mismatch rate was again approximately 10%, suggesting that *O. melastigma* might be divergent from *O. latipes*.

The core eukaryotic genes (CEGs) [25] are highly conserved, present in all eukaryotic species and found in low numbers of in-paralogs in different species. A majority of the CEGs are expected to be present in a quality transcriptome assembly. Among the 248 CEGs, 99.6% were recovered in the “Reads-combined Assembly” (see below), and the average e-value was highly significant (2.05E-14) and average percentage identity of the matched transcripts were 95.9% (details in Additional file 6: Table S6).

When comparing the assembled transcripts with known *O. melastigma* mRNA sequences using BLASTn and known *O. melastigma* protein sequences using BLASTx, we found that 92.4% (327/354) of known transcripts were recovered in the Reads-combined Assembly, while 86.4% (323/374) of the known *O. melastigma* protein sequences were recovered, suggesting our assembly should be largely complete. However, some tissue-enriched genes in organs other than the brain, liver, ovary and testis may have been missed (details in Additional file 7: Table S7, Additional file 8: Table S8, Additional file 9: Table S9).

To aid in the comparison of gene expression among different *O. melastigma* tissues, we explored two approaches to generate a single consensus transcriptome assembly; (1) Reads-combined Assembly: sequence reads for all tissues were combined prior to being subjected to *de novo* assembly [23] and (2) Contigs-clustered Assembly: assembly was performed individually for each library. Redundant transcripts were identified, and representative transcripts were chosen by clustering [21,37]. When comparing the two approaches, the Reads-combined Assembly recovered more CEGs than the Contigs-clustered Assembly. More importantly, the Reads-combined Assembly had significantly more RBHs than the Contigs-clustered Assembly (14,628 vs 12,145). Moreover, the average contig length (1302 bp vs 1086 bp) and N50 (2908 bp vs 2450 bp) was longer for the Reads-combined Assembly. Taken together, we believe the Reads-combined Assembly represents a more complete consensus transcriptome assembly for inter-organ comparison.

### Protein-coding genes expression in the brain, liver, ovary and testis of O. melastigma

Protein-coding ORF prediction followed by Reciprocal Best Hit BLAST resulted in 14,628 annotated genes that were found across the brain, liver and gonadal tissues of *O. melastigma* (Table 1). The highest numbers of annotated genes were expressed in brain tissue. In females, 14240, 9200 and 13240 annotated genes were identified in brain, liver and ovary, respectively. In males, 13,796, 10,763, and 13,618 annotated genes were identified in the brain, liver and testis, respectively. For brain tissue, the female- and male- combined assembly improved the assembly slightly and yielded 14,267 annotated genes. For liver tissue, the female- and male- combined assembly significantly improved the discovery and resulted in 11,438 annotated genes.

**Table 1:** **Number of genes identified in different organs of** ***O*** **.** ***melastigma***

**Organ**	**Gender**	**Number of identified genes**
Brain	Female	14,240
	Male	13,796
Liver	Female	9,200
	Male	10,763
Ovary	Female	13,240
Testis	Male	13,618
Total number	/	14,628

### Tissue-enriched genes in O. melastigma

**Figure 3:**
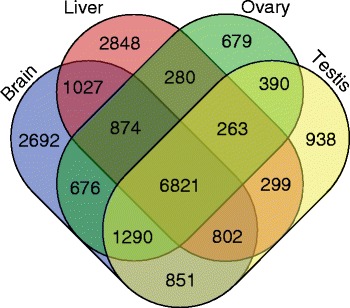
**Global comparison of annotated genes in the brain, liver, ovary and testis of marine medaka.** Of the identified genes, 2692 were brain-enriched. 2848 genes were liver-enriched, and 2007 genes were gonad-enriched. 6821 annotated genes were common to all tissues in both males and females.

Global comparison of annotated genes showed that 7157 (34.5%) genes that were annotated in only a single tissue. We found 2692 brain-enriched genes, while 2848 genes were liver-enriched, and 2007 genes were gonad-enriched. Furthermore, 6821 annotated genes were common to all tissues in both males and females (Figure 3). The gonad-enriched genes were enriched in the following GO terms: sexual reproduction and gamete generation. The brain-enriched genes were enriched in functions related to channel activity, synaptic transmission and cell-cell adhesion. The liver-enriched genes were enriched in functions related to metabolic processes, transferase and mannosidase activity (Table 2, Additional file 10: Table S10).

**Table 2:** **Functional enrichment of Gene Ontology terms in organ-enriched genes**

	**Gene Ontology**	**ID**	**Category**	**Benjamini & Hochberg corrected p-value**
**Gonad-enriched genes common to male and female marine medaka**	*sexual reproduction*	GO:0019953	Biological Process	4.42E-04
	*gamete generation*	GO:0007276	Biological Process	4.42E-04
**Brain-enriched genes**	*gated channel activity*	GO:0022836	Molecular Function	8.81E-10
	*signaling*	GO:0023052	Biological Process	2.98E-06
	*transmission of nerve impulse*	GO:0019226	Biological Process	8.35E-06
	*potassium channel activity*	GO:0005267	Molecular Function	8.80E-06
	*cell*-*cell adhesion*	GO:0098609	Biological Process	4.92E-05
	*nervous system development*	GO:0007399	Biological Process	4.20E-05
	*synapse*	GO:0045202	Cellular Component	2.25E-04
**Liver-enriched genes**	*cellular macromolecule metabolic process*	GO:0044260	Biological Process	1.57E-12
	*RNA metabolic process*	GO:0016070	Biological Process	3.34E-07
	*nitrogen compound metabolic process*	GO:0006807	Biological Process	4.62E-07
	*transferase activity*	GO:0016740	Molecular Function	1.09E-05
	*protein modification process*	GO:0036211	Biological Process	2.77E-03
	*kinase activity*	GO:0016301	Molecular Function	2.87E-03
	*mannosidase activity*	GO:0015923	Molecular Function	1.10E-02

**Figure 4:**
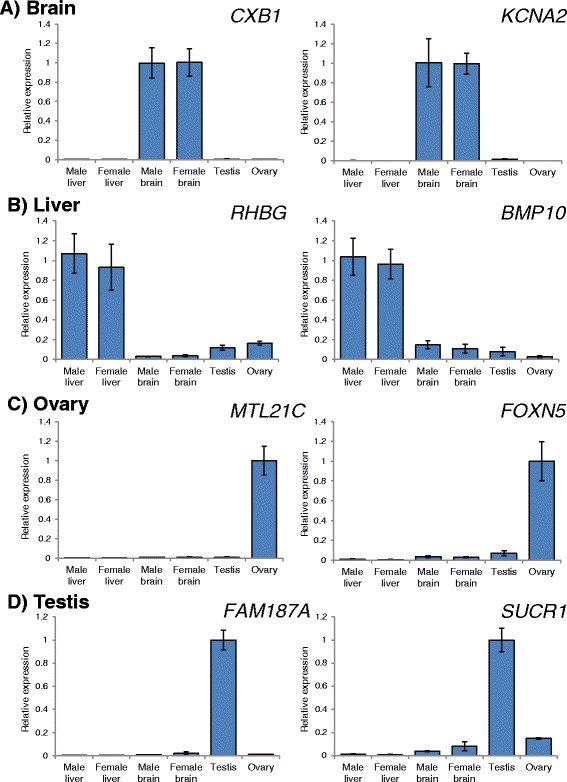
**qPCR validation of tissue-enriched genes in marine medaka. A)** Specific expression of gap junction beta-1 protein (*CXB1*) and potassium voltage-gated channel, shaker-related subfamily, member 2 (*KCNA2*) in the female and male brain. **B)** Dominant expression of Rh Family, B Glycoprotein (*RHBG*) and bone morphogenetic protein 10 (*BMP10*) in the liver compared to other tissues. **C)** Specific expression of methyltransferase-like 21C (*MTL21C*) and dominant expression of forkhead box protein N5 (*FOXN5*) in the ovary. **D)** Dominant and specific expression of family with Sequence similarity 187, member A (*FAM187A*) and succinate receptor 1 (*SUCR1*) in the testis.

We identified the tissue-enriched genes using a more conservative read-count approach. The expression of tissue-enriched genes was validated using qPCR analysis. Some of the genes were closely related to the functions of corresponding tissues. Our results demonstrated that gap junction beta-1 protein (*CXB1*) and potassium voltage-gated channel subfamily A member 2 (*KCNA2*) were highly expressed in both male and female marine medaka brain tissue (Figure 4A). Gap junction protein is the major component of gap junction channels that controls the exchange of ions and small molecules between cells. In the human brain, *CXB1* is highly expressed in neurons and oligodendrocytes and appears to be critical for the functions of Schwann cells, which are responsible for the myelination of nerves in the peripheral nervous system [38,39]. *KCNA2* is present in most voltage-gated ion channels and plays important biological functions in the brain, including neurotransmitter release and neuronal excitability. Knockdown of *KCNA2* reduces the total voltage-gated potassium current, resulting in increased excitability in neurons and neuropathic pain symptoms in rats [40]. The identification of genes related to brain functions could largely facilitate the use of marine medaka as an *in vivo* model for neuro-toxicological studies.

Our qPCR analysis also indicated high expression of ammonium transporter Rh type B (*RhBG*) and bone morphogenetic protein 10 (*BMP10*) in the marine medaka liver (Figure 4B). Studies in mice have demonstrated that RhBG is highly expressed in the perivenous hepatocytes of the liver, which is an important tissue for ammonium metabolism and mediates ammonium uptake [10,12]. *BMP10* is a member of the transforming growth factor β (*TGF β*) superfamily, whose members interact with membrane-bound receptors to exert their biological functions [41]. Analysis of *BMP10*-deficient mice demonstrated that *BMP10* has an exclusive function in early cardiac development [42]. However, its function in the liver is still elusive. We also found an elevated level of methyltransferase-like 21C (*MTL21C*) and forkhead box protein N5 (*FOXN5*) in the ovaries of marine medaka (Figure 4C). *MTL21C* is a newly identified lysine methyltransferase that regulates the activities of various molecular chaperones, as well as the lysine residues in heat shock protein 70 [43]. Studies in pigs have demonstrated that heat shock chaperones play an important role in thermal stress adaptation [44]. *FOXN5* is Forkhead-box (FOX) gene which is implicated in embryogenesis through transcriptional regulation. Study in mouse demonstrated that germ-line mutation of *FOXN5* gene in the mouse lineage might lead to divergent scenario of early embryogenesis through the deregulation of *FOXN5* target genes in mouse early embryos [45,46]. Last, our result demonstrated that succinate receptor 1 (*SUCR1*) and the Ig-like V-type domain-containing protein FAM187A (*FAM187A*) were highly expressed in marine medaka testicular tissues (Figure 4D). In humans, *SUCR1* is expressed in a variety of tissues, including adipose, liver, and kidney tissue [47]. This protein is a G protein-coupled receptor that senses cellular stresses such as hypoxia, toxicity, and hyperglycemia. Taken together, our results identified a number of tissue-enriched genes in the brain, liver, testis and ovary of marine medaka and may largely facilitate the use of *O. melastigma* for marine ecotoxicological studies at the organ level.

### Marine-to-freshwater orthologous transcripts and marine-enriched transcripts

To compared the conservativeness between marine medaka (*O. melastigma*) and freshwater medaka (*O. latipes*), Reciprocal Best Hit BLASTn was used. We estimated *O. melastigma* and *O. latipes* had 5880 orthologous protein-coding transcripts, requiring more than 70% length recovery of *O. latipes* transcripts (Additional file 11: Table S11).

The capability of animal cells to maintain a constant cell volume is prerequisite for cellular life. When eukaryotic cells are exposed to extracellular osmotic stress, they undergo rapid regulatory processes to maintain their cellular homeostatic status. The mechanism is particularly important in gill epithelia in fishes. Here, we showed the RNA-seq data from two medaka fishes that live in different osmotic environments. *O. melastigma* inhabits in brackish-water or fresh water around Begal Bay and Malay Peninsula; while *O. latipes* are found in fresh water of Japan, Korea and China. They encounter different osmotic environments and have been shown to have different osmotic tolerances in fresh water to seawater transfer experiments [48]. In a molecular point of view, the two fishes should have different osmoregulatory mechanisms. In fish biology, we know that the gill is the first osmoregulatory tissue to sense and response the osmotic challenges [49]. In addition, kidney and intestine play osmoregulatory roles in fish [50,51]. Although our transcriptome data of *O. melastigma* do not include the osmoregulartory tissues/organs, our data have identified several critical seawater acclimating ion transporters, such as cystic fibrosis transmembrane conductance regulator, sodium/potassium/chloride co-transporter, and sodium pump α and β. These ion transporters have been shown to be highly expressed in gills of SW acclimated fishes, such as eel, and tilapia [52-54]. The identification of these ion transporters in the *O. melastigma* suggested the possible use of our RNA-seq data for future osmoregulatory studies.

**Figure 5:**
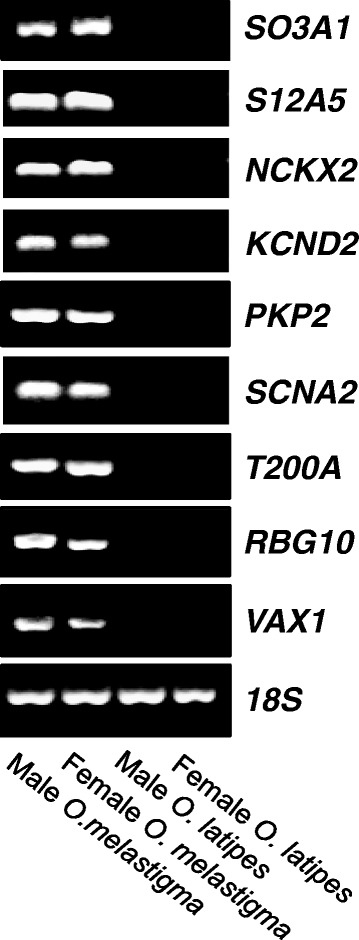
**Validation of** ***O*** **.** ***melastigma*** **-** ***enriched*** **genes by RT-PCR.** The marine medaka specific genes were validated in both male and female marine medaka against freshwater medaka.

Furthermore, by using read-count approach and qPCR validation, we estimated that a lower boundary of 255 genes being only expressed in *O. melastigma* compared to those in the *O. latipes* database (Additional file 12: Table S12). The highly expressed genes in *O. melastigma* and some selected genes that might be functionally related to seawater adaptation were further validated by RT-PCR. Indeed, our results showed that a number of genes were highly expressed in *O. melastigma* but undetected in *O. latipes* (Figure 5). One of the *O. melastigma*-enriched genes, solute carrier and organic anion transporter (*SO3A1*), is commonly found in human brain tissue and epidermal keratinocytes. *SO3A1* may play a role in the exchange of anions between cells, thus facilitating seawater adaptation [55]. In addition, it mediates the transport of thyroxine and vasopressin [56] that is important in osmoregulation [57,58]. Similarly, another solute carrier, solute carrier family 12 member 5 (*S12A5*), is commonly found in brain. It is a potassium-chloride co-transporter, which is highly expressed in neurons [59]. In addition, the sodium-calcium-potassium exchanger 2 (*NCKX2*) is a polytopic membrane protein that drives Ca^2^+ extrusion across the plasma membrane [60]. All these three transporters mentioned above are highly expressed in the brain region. However, they all cannot be aligned in the recent existing freshwater medaka. Additionally, cardiac channels such as potassium voltage-gated channel subfamily D member 2 (*KCND2*) and plakophilin-2 (*PKP2*) also only be found in the marine medaka. *KCND2* is critical in repolarizing the cardiac action potential [61], while *PKP2* is essential protein for building up of desmosome. *PKP2* has been reported to be functionally related to sodium channel, and decreased in *PKP2* expression leaded to downregulation of sodium current in cardiomyocytes of human [62,63]. The data presented here, hence provides opportunities for researchers to understand the ion transporters mechanism between two species by using our database as nucleotide references for different molecular probes.

Nevertheless, instead of using the existing model organism genome database, our findings suggest that researchers generate their own model transcriptome database for a more confident result. Even in the two close species we examined here, there are genes that cannot be aligned in the seawater medaka. It should be noted that some seawater-unique genes we mentioned above are common in different species; the reason that we cannot identify them in the freshwater medaka may due to their low similarities between the two species. In fact, the ambient conditions, age, and the physiological state when tissue samples were collected influences the transcription rate of a gene, and whether or not a gene is expressed at all. We also note that allelic variation might explain the observed large divergence between the orthologous transcripts between marine and freshwater medaka. Nevertheless, this further supports the necessity of generating species-specific database for ecotoxicological studies.

## Conclusion

This study provides a specific marine medaka transcriptome resource to the community that could facilitate future works on the marine medaka. We annotated more than 14,000 transcripts across four tissues in marine medaka and found 5880 orthologous transcripts between *O. melastigma and O. latipes*. Moreover, numerous tissue/organ-enriched genes were identified. Most importantly, we further investigated the possible divergence between *O. melastigma* and *O. latipes*, which suggests the importance of generating the model's own transcriptome database. This sequencing effort generated a valuable resource of coding DNA for a non-model species that will aid future studies assessing *in vivo* molecular responses to environmental stresses and biological toxicity in the marine environment.

### Availability of supporting data

The sequence data from this study have been submitted to the NCBI Sequence Read Archive (SRA) (http://www.ncbi.nlm.nih.gov/sra) under the accession number SRP041838. The assembled transcripts were deposited in at DDBJ/EMBL/GenBank under the accession GBFY00000000; GBFX00000000; GBFW00000000; GBFV00000000; GBFU00000000; GBFT00000000; GBFS00000000; GBFR00000000; GBFQ00000000 and GBGE00000000 under BioProject ID: 246584.

## Additional files

Additional file 1: Table S1. qPCR Primer list. Primer list used in qPCR validation. Additional file 2: Table S2. Sequencing summary. Summary of Illumina high-throughput sequencing. Additional file 3: Table S3. Mapping statistics for freshwater medaka. Reference mapping of the *O. melastigma* RNA-Seq data generated in this study onto the freshwater medaka genome and UniGENE database. Additional file 4: Table S4. Transcriptome *de novo* assembly statistics. Technical statistics of the *de novo* transcriptome assembly. Additional file 5: Table S5. Identity between the *Oryzias latipes* UniGENE dataset and *Oryzias melastigma* assembled transcripts. Comparison between the *O. latipes* UniGENE dataset and *O. melastigma* assembled transcripts reveals divergent transcriptomes in terms of nucleotide identity. Additional file 6: Table S6. Recovery of eukaryotic core genes in the assembled transcriptome. Recovery of the 248 eukaryotic core genes in the assembled transcriptome. Additional file 7: Table S7. Summary of the recovery of known *O. melastigma* sequences in the assembled transcriptome. Summary of the recovery of known *O. melastigma* sequences in the assembled transcriptome. Additional file 8: Table S8. Details of the recovery of known *O. melastigma* mRNA sequences in the assembled transcriptome. Details of the recovery of known *O. melastigma* mRNA sequences in the assembled transcriptome. Additional file 9: Table S9. Details of the recovery of known *O. melastigma* protein sequences in the assembled transcriptome. Details of the recovery of known *O. melastigma* protein sequences in the assembled transcriptome. Additional file 10: Table S10. Gene Ontology enrichment for the tissue-enriched genes. Gene Ontology enrichment for the brain-, liver- and gonad-enriched genes. Additional file 11: Table S11. Orthologus transcripts between *O. melastigma* and *O. latipes*. Additional file 12: Table S12.
*O. melastigma*-enriched transcripts. Marine medaka *O. melastigma* transcripts that are not expressed in freshwater medaka *O. latipes*.

## Abbreviations

(*O. melastigma*): *Oryzias melastigma*
(*O. latipes*): *Oryzias latipes*
(ORFs): Open Reading Frames
(RBH): Reciprocal Best Hit
(CEG): Core Eukaryotic Genes
(SRA): Sequence Read Archive
